# Caffeine mitigates ROS accumulation and attenuates motor neuron degeneration in the wobbler mouse model of amyotrophic lateral sclerosis

**DOI:** 10.1186/s12964-025-02415-5

**Published:** 2025-09-10

**Authors:** Aimo Samuel Christian Epplen, Maximilian Rothöft, Sarah Stahlke, Carsten Theiss, Veronika Matschke

**Affiliations:** 1 Department of Cytology, Institute of Anatomy, Medical Faculty, Ruhr-University Bochum, Universitätsstr. 150, Building MA 5/52, Bochum, 44801 Germany; 2 International Graduate School of Neuroscience (IGSN), Ruhr-University Bochum, Bochum, Germany; 3 Medical Imaging Center (MIC), Electron Microscopy Medical Analysis - Core Facility (EMMA CF) Medical Faculty, Ruhr-University Bochum, Bochum, Germany

**Keywords:** ALS, Neurodegenerative disease, Reactive oxygen species (ROS), Antioxidative defense, NAD, Muscle strength

## Abstract

**Background:**

Amyotrophic lateral sclerosis (ALS) is a devastating neurodegenerative disease characterized by oxidative stress and progressive motor neuron degeneration. This study evaluates the potential neuroprotective effects of caffeine in the Wobbler mouse, an established model of ALS.

**Methods:**

Wobbler mice received caffeine supplementation (60 mg/kg/day) via drinking water, and key parameters, including muscle strength, NAD metabolism, oxidative stress, and motor neuron morphology, were assessed at critical disease stages.

**Results:**

Caffeine delayed motor performance decline, as observed in grip strength tests during the early symptomatic phase. Histological analyses revealed that significantly fewer motor neurons were lost in caffeine-treated mice at p41, despite no changes in soma morphology. Biochemical assays demonstrated that caffeine significantly reduced ROS levels and restored NAD levels to wildtype-like values, although NMNAT2 protein expression remained unaffected. The data suggest that caffeine mitigates oxidative stress through alternative pathways, potentially involving enhanced mitochondrial function and antioxidative defenses.

**Conclusions:**

These findings highlight the potential of caffeine as a protective agent for delaying motor neuron degeneration in ALS. Future studies should explore optimal dosing strategies, combinatorial treatment approaches, and the underlying molecular mechanisms, to enable translation of these findings to human ALS patients.

**Supplementary Information:**

The online version contains supplementary material available at 10.1186/s12964-025-02415-5.

## Background

Amyotrophic lateral sclerosis (ALS) is a lethal neurodegenerative disease with a prevalence ranging from 1.6 to 11.8 per 100,000 globally [1]. Familial (fALS, 10%)and sporadic (sALS, 90%) forms exist [2], yet no definitive therapy is currently available. Riluzole remains the only disease-modifying treatment for amyotrophic lateral sclerosis (ALS) approved in Germany, and is widely used internationally. While randomized controlled trials originally suggested a modest extension of survival by 2–3 months, real-world evidence from clinical databases across Europe, North America, and Asia has shown that Riluzole can extend median survival by up to 19 months, particularly when initiated early in the disease course [3–5]. Edaravone, approved in Japan, South Korea, and the USA, is thought to reduce oxidative stress, though its exact mechanism remains unclear [6]. Despite extensive research, most investigational substances have failed in clinical trials, leaving no causative therapy to date [6–8].

Oxidative stress is a hallmark of ALS, particularly in sporadic forms. Elevated levels of reactive oxygen species (ROS) have been detected in various samples from ALS patients, causing damage to proteins, lipid membranes, and DNA [9–12]. This cellular damage is well documented across ALS models [13–18], highlighting oxidative stress as a central driver of disease progression.

The Wobbler mouse serves as a well-established model for studying ALS pathomechanisms and evaluating therapeutic strategies. This model is due to an autosomal recessive mutation in the vacuolar protein sorting-associated protein (Vps54) gene [19] affecting Golgi-associated retrograde protein (GARP) complex [20] and leading to impaired vesicle transport, protein aggregation, and motor neuron degeneration [19, 21, 22]. Phenotypically, Wobbler mice exhibit progressive ALS-like symptoms, including unsteady gait, muscle atrophy, and respiratory failure [23–26]. From birth, Wobbler mice develop similarly to their healthy wildtype siblings for approximately three weeks. Around postnatal day 20 (p20), the first symptoms, such as unsteady gait, head tremor, and muscle atrophy, become evident, marking the early symptomatic stage. As the disease progresses, animals enter a stable clinical phase around postnatal day 40 (p40), during which symptoms worsen, and motor neuron degeneration intensifies [23, 24]. Notably, elevated levels of oxidative stress, such as increased reactive oxygen species (ROS) in the spinal cord [27] and specifically motor neurons [28], become detectable only during this phase. Moreover, oxidative stress-related damage, such as to DNA, proteins, and organelles, has been demonstrated in various ALS mouse models, including the Wobbler mouse [28–33].

Given the shared feature of increased oxidative stress in ALS patients and the Wobbler mouse, this study explores the potential of caffeine as a therapeutic agent. Caffeine is widely recognized for its antioxidant and neuroprotective effects, including its ability to reduce biomarkers of oxidative stress and lower the risk of neurodegenerative diseases such as Parkinson’s disease [34, 35]. These effects are commonly attributed to both direct radical scavenging [36] and the activation of endogenous antioxidant systems. In particular, caffeine has been shown to stimulate the NRF2 signaling pathway [37–39], thereby enhancing the expression of key antioxidant enzymes such as SOD, CAT, and GPx [40–42]. It mitigates oxidative DNA damage, as demonstrated in rat lungs [43], by neutralizing hydroxyl free radicals and inhibiting lipid peroxidation [36, 44]. Beyond these effects, caffeine has been shown to enhance levels of nicotinamide mononucleotide adenylyltransferase 2 (NMNAT2), a key enzyme in NAD metabolism, in neuronal cells [45] and to restore NMNAT2 levels in Wobbler motor neurons [27, 46]. NMNAT enzymes (NMNAT1-3) are crucial for axonal development and survival [47], with NMNAT2 predominantly located in the cytosol and Golgi apparatus of brain cells [45, 48, 49]. Beyond this metabolic role, NMNAT significantly supports cellular antioxidant defense systems by regulating ROS levels and influencing cell fate decisions [50]. These properties make caffeine a promising candidate for addressing oxidative stress and promoting neuronal resilience in ALS.

The aim of this study is to investigate the effects of caffeine in vivo in the Wobbler mouse, a naturally occurring model of ALS caused by a spontaneous mutation in the Vps54 gene. Our specific focus lies on its impact on NAD^+^ homeostasis via NMNAT2, based on our previous in vitro findings showing that caffeine enhances *Nmnat2* expression and restores NAD⁺ levels in cultured Wobbler motor neurons, leading to improved neurite outgrowth. Unlike transgenic overexpression models, the Wobbler mouse offers a complementary, non-transgenic approach to studying ALS pathophysiology and potential interventions. Key objectives of this study include the assessment of muscle strength, as well as the analysis of cervical spinal cord tissue for changes in NMNAT2 protein expression, NAD⁺ levels, and oxidative stress levels. Additionally, motor neuron morphology is evaluated to gain histological insights. To comprehensively capture the dynamic changes during disease progression, we examine three critical time points: postnatal day 20 (p20), representing early symptomatic onset; p26, a transitional phase with developing symptoms; and p41, the stable clinical stage characterized by pronounced oxidative stress. While previous studies highlight the neuroprotective potential of caffeine in various models of neurodegeneration, our study provides the first systematic in vivo analysis of both functional and molecular effects of caffeine in the Wobbler mouse across multiple disease stages, offering novel insights into its therapeutic potential in ALS.

## Methods

### Animals

#### General information

All animal procedures were conducted in strict compliance with the guidelines of the German federal state of North Rhine-Westphalia and the European Communities Council Directive 2010/63/EU for the protection of animals used for scientific purposes. The animal experiment “Investigation of the motor performance of Wobbler mice after pharmacological treatment” (project number 81-02.04.2020.A375) was approved by the competent authority and carried out in accordance with its specifications. Transcardial perfusion was separately approved under the project number 84-02.04.2017.A085.

The study utilized C57BL/Fa mice with the Wobbler mutation (homozygous WR) and wildtype mice (homozygous WT) as controls, while heterozygous mice were used for breeding purposes. Breeding and genotyping followed previously described protocols [23]. All mice were housed under a 12-hour light/dark cycle in open cages with *ad libitum* access to food and water, or water supplemented with caffeine.

A total of 64 mice (both male and female) were used for the strength tests: 32 WR and 32 WT. Each genotype was evenly divided into control and caffeine-treated groups, resulting in four groups of 16 animals: WT Control, WT Caffeine, WR Control, and WR Caffeine (hereafter referred to as WT, WT Caf, WR, and WR Caf, respectively). To investigate age-dependent development in the analysis, tissue samples were collected from animals at postnatal day 20 (p20), day 26 (p26), and day 41 (p41). Both sexes were included in all experimental groups. Preliminary analyses revealed no significant sex-specific differences in the assessed parameters. Thus, data from male and female mice were pooled for all subsequent analyses.

#### Caffeine supplementation

In the control group, test animals received complete feed and water *ad libitum*. Mice in caffeine group ingested caffeine through supplemented drinking water, which was freely available for a period of three weeks (p20-p41). The caffeine dosage was set at 60 mg/kg/day, a concentration well-documented in previous studies [51, 52]. This dose in mice corresponds to a human equivalent dose of approximately 340 mg per day, comparable to the caffeine content of about 3–4 cups of regular coffee in humans. Fresh caffeine solutions were prepared every three days using caffeine powder (Sigma-Aldrich, Cat# 1.02584). At postnatal day 20 (p20), the animals were separated from the parent animals and housed in cages with either caffeine-supplemented or regular drinking water. To ensure that animals of both genotypes (WT and WR), despite differing body weights, received a comparable caffeine dose, the concentration of caffeine in drinking water was set to 0.36 mg/ml. This concentration was determined based on expected drinking volumes per animal and body weight progression from p20 to p40 [53, 54]. According to our prior observations and published data, WR mice consumed slightly less water than WT mice and showed lower body weight throughout the experimental period. Supplementary Table S1 summarizes body weight, estimated drinking volumes, and the resulting range of caffeine doses (mg/kg/day), confirming that the target dose of 60 mg/kg/day was consistently achieved or slightly exceeded.

**Fig. 1:**
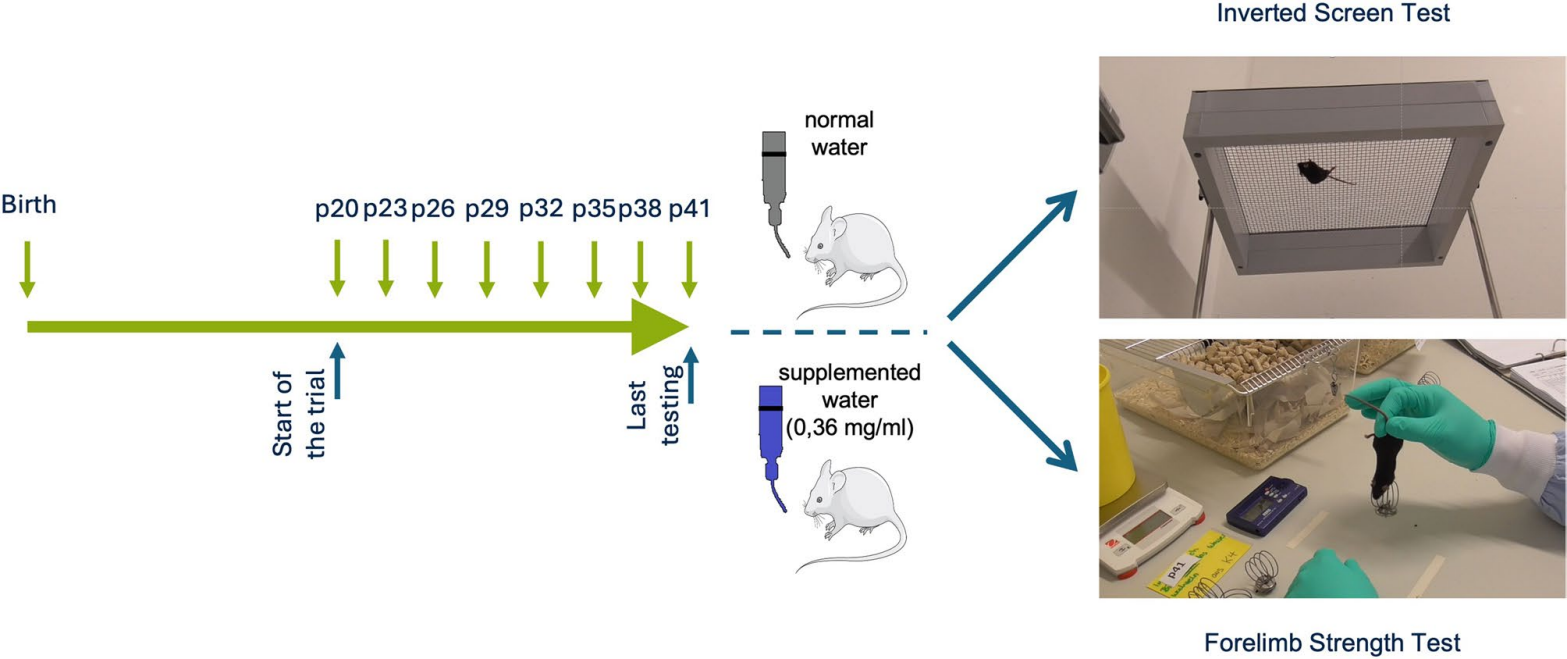
Experimental timeline and muscle strength assessment in Wobbler mice. The study timeline illustrates the experimental setup from birth to the last testing point at postnatal day (p)41. Caffeine supplementation (0.36 mg/ml) was administered via drinking water from p20 onwards, while control mice received normal water. Based on average water intake, this concentration corresponds to an estimated daily caffeine consumption of ~ 60 mg/kg body weight/day. Muscle strength was evaluated at multiple time points (p20–p41) using two complementary tests: the Inverted Screen Test (top right), which assesses grip strength of all four limbs, and the Forelimb Strength Test (bottom right), which measures forelimb-specific strength using weighted handles

To monitor the effects of caffeine treatment on muscle strength, all animals were subjected to two muscle strength tests every three days: (i) Assessment of muscle strength using all four limbs: Inverted Screen Test, and (ii) Forelimb Strength Measurement (Fig. 1). These tests allowed for a comprehensive evaluation of all four limbs and forelimb-specific strength during the treatment period.

#### Evaluation of muscle strength

##### Assessment of whole-body grip strength: inverted screen test

The inverted screen test, first described by Kondziela in 1964, is a standard method for assessing the muscle strength of mice across all four limbs simultaneously [55]. The test uses a square wire mesh (40 × 40 cm, 1 mm wire thickness, grid squares 12 × 12 mm), equipped with an ∼8 cm high frame to prevent the mouse from climbing over the edge [56]. For this study, a modified setup was implemented to ensure standardized and objective testing (Fig. 1). The wire grid with its frame (hereinafter referred to as the “grid”) is rotatably mounted on a bipod, allowing rotation around a central axis. A mechanical stop Limits the rotation to 180°, enabling fixation in either the “mouse up” or “mouse down” position. A padded box beneath the apparatus ensures that falling mice do not sustain injuries.

Mice were acclimatized to the test environment for 15–20 min before testing. After each trial, mice were returned to their cages for recovery. During testing, each mouse was placed at the center of the grid. The grid was positioned at a height of 45 cm to ensure consistent testing conditions. The grid was then inverted forward over the mouses head, and the time the mouse could maintain its grip was measured, with a maximum duration of 60 s. Healthy mice typically hold on for the full minute without difficulty [56]. Each mouse underwent three trials per test day to assess maximum grip strength, with a minimum recovery time of 60 s between trials. The highest recorded duration from the three trials was used as the measure of maximum force for each mouse.

A total of 64 mice (16 animals per group) were examined in this study. The results were based on the maximum duration of grip strength recorded for each mouse during the test.

##### Forelimb strength measurement

To assess the strength of the forelimbs only, mice were held by the base of the tail and allowed to grasp a weight handle consisting of wire and variable numbers of washers using both forepaws (Fig. 1). The following weights were used: 5, 9, 13, 17, 20, 30, 40 and 50 g.

During the test, the mouse was held at the base of the tail, suspended upside down, and brought close to the handle. The mouse instinctively grasped the handle, Lifting the weight off the table when raised. A successful trial was defined as the mouse holding the weight for three seconds. After a pause of at least 10 s, the next heavier weight was presented. If the mouse dropped the weight, the same weight was retested following a recovery period. If the mouse failed to hold the weight for three seconds on three consecutive attempts, the test was concluded, and the maximum successfully held weight was recorded as the final result. To standardize timing and ensure that the test could be conducted objectively by a single observer, a metronome was used to provide a precise second cycle for measuring the three-second holding duration.

To quantify forelimbs strength, a scoring system was developed that incorporates both the maximum successfully held weight and the duration for which the subsequent heavier weight was held. For example, if a mouse successfully held 20 g for three seconds but held 30 g only for two seconds, the score was calculated as 20 + 2 = 22. If the mouse was unable to Lift 30 g but held 20 g for three seconds, the score was 20. This scoring system ensures precise and reproducible quantification of forelimb strength and facilitates clear comparisons between experimental groups.

### Measurement of oxidative stress

Oxidative stress in the cervical spinal cord was evaluated using the Superoxide Anion Microplate Assay Kit (Biorbyt, Cambridge, United Kingdom, Cat# orb545630) according to the manufacturer’s protocol. After decapitation, the cervical spinal cord was isolated, weighed, snap-frozen in liquid nitrogen, and stored at −80 °C until further use. For this study, tissue from 6 WT and 6 WR at p20, as well as 4 animals per group (control or caffeine-treated) at p26 and p41 were used. To prepare the samples, 100 mg of tissue was homogenized in 1 ml of assay buffer. Homogenates were centrifuged at 8,000 g for 10 min at 4 °C, and the resulting supernatant was collected for analysis. Substrate and two dye reagents were added to the samples according to the manufacturer’s instructions. Samples were incubated for 20 min at 37 °C, and absorbance was measured at 530 nm in duplicate. All measured values were normalized to the mean of untreated WT samples to enable comparison across groups. The normalized data were presented as a bar chart for visualization.

### NMNAT2 protein-expression analysis via western blot

NMNAT2 protein expression was analyzed using Western Blot. Protein extraction from cervical spinal cord tissue was performed with RIPA buffer (150mM NaCl, 20mM Tris-HCl, 0.1% sodium dodecyl sulfate, 1% Triton-X100, 1% sodium deoxycholate, 1mM Na2EDTA) supplemented with 1% cOmplete EDTA-free protease inhibitor (Roche Diagnostics, Mannheim, Germany, Cat# 05056489001). Tissue samples were kept on ice and homogenized with a micro pestle in 10 µl of lysis buffer per mg of tissue. The homogenates were centrifuged at 11,000 g for 10 min at 4 °C, and the supernatants were collected for analysis. Protein concentrations were determined using the Pierce™ BCA Protein Assay Kit (Thermo Fisher Scientific, Waltham, MA, USA, Cat# 23227). For each sample, 50 µg of total protein was loaded per lane and separated by SDS-Page. Proteins were transferred onto nitrocellulose membranes (TransBlot Turbo Mini-Size Nitrocellulose, #1704158, Biorad, Hercules, CA, USA). Blocking was carried out at room temperature for at least 1 h using either 10% RotiBlock in 1xTBS (#A151, Roth, Karlsruhe, Germany, for Calnexin as loading control) or 5% milk powder in 1xTBS (for NMNAT2). Detection of NMNAT2 was performed using a primary mouse monoclonal IgG anti-NMNAT2 antibody (1:200 dilution in 1xTBS; #sc-515206, Santa Cruz Biotechnology, Dallas, TX, USA) and a secondary peroxidase-conjugated horse anti-mouse antibody (1:10,000; #PI2000, Vector Laboratories Inc., Newark, CA, USA). For detection of calnexin, a primary rabbit polyclonal IgG anti-calnexin antibody (1:1,000 dilution; #NB100-1965, Novus Biologicals, Littleton, CO, USA) and a secondary peroxidase-conjugated goat anti-rabbit antibody (1:10,000 dilution; #PI1000, Vector Laboratories Inc., Newark, CA, USA) were used. Protein signals were detected using Clarity Western ECL Substrate (#170–5061, Biorad, Hercules, CA, USA). Band intensities were quantified with Image Lab Software (Version 6.1, Biorad, Hercules, CA, USA). Data were normalized to calnexin, and the normalized values of Wobbler samples were compared to normalized wildtype samples at each developmental stage. A total of *n* = 4 animals per group were analyzed.

### NAD^+^ assay

To measure NAD^+^ levels in spinal cord tissue at stages p20, p26 and p41 in both control and caffeine-treated wildtype and Wobbler mice, the NAD/NADH-Glo Assay (#G9071, Promega, Madison, WI, USA) was used according to manufacturer’s protocol. In brief, 10 mg of spinal cord tissue were lysed in 500 µl lysis buffer (1:1 mixture of 1xPBS and 0.2 N NaOH + 1% (v/v) DTAB) at room temperature. For NAD^+^ measurement, 100 µl of each lysate was mixed with 50 µl of 0.4 N HCl solution, incubated at 60 °C for 15 min, and subsequently cooled to room temperature for 10 min. Neutralization was achieved by adding 50 µl of 0.5 M Trizma-base solution. Each sample (50 µl) was placed in duplicate in a 96-well-plate (82.1581.110, Sarstedt, Nümbrecht, Germany.) with 50 µl of detection reagent. Following a 30-min incubation, luminescence was measured using a SpectraMax iD3 multi-mode microplate reader (Molecular Devices, San Jose, CA, USA) with a measurement time of 0.25s. The obtained data were normalized to the protein content of each sample, determined using the Pierce BCA Assay (#23225, Thermo Fisher Scientific, Waltham, MA, USA). A sample size of *n* = 4 was used for each group.

### Morphological analysis of motor neurons

To assess the effects of the treatment on motor neuron structure and organization, the number and morphology of motor neurons in the cervical spinal cord was quantified. Mice were deeply anesthetized using a combination of ketamine (100 mg/kg) and xylazine (10 mg/kg) to enable transcardial perfusion. Perfusion was carried out with 2.5% glutaraldehyde (#G5882, Merck, Darmstadt, Germany) in phosphate buffer. The tissue was post-fixed in Dalton solution (1 g OsO4; [#19134, Electron Microscopy Sciences, Belgium] dissolved in 100 ml 5% potassium dichromate solution [#7953, Roth, Germany]) for 2 h, then washed in phosphate buffer. The tissue was dehydrated through an ascending ethanol series starting in 50%, followed by incubation in 70% ethanol containing 1% uranyl acetate (#21447, Polyscience Inc., UK) and 1% phosphotungstic acid (#455970, Merck, Darmstadt, Germany) overnight at 4 °C. Dehydration was completed with ethanol concentrations up to 100%. The specimens were then infiltrated with epoxy resin through incubation in propylene oxide (#807027, Merck, Darmstadt, Germany) followed by ascending series of propylene oxide and EPON mixtures (3:1, 1:1, 1:3 ratios). EPON consisted of glycidether (#21045.02, Serva Electrophoresis GmbH, Heidelberg, Germany), methylnadic anhydride (#29452.02, Serva Electrophoresis GmbH, Heidelberg, Germany), 2-dodecylsuccinic acid anhydride (#20755.01, Serva Electrophoresis GmbH, Heideleberg, Germany) and 2,4,6-tris(dimethylaminomethyl)phenol (#36975.01, Serva Electrophoresis GmbH, Heidelberg, Germany) in a 5.4 : 3.8 : 1.84 : 1 ratio. The specimens incubated with pure EPON overnight at 20 °C and polymerized at 60 °C for 2d. Semi-thin Sect. (0.5 μm thick) were prepared using an Ultracut E Reichert-Jung (Leica Microsystems GmbH, Wetzlar, Germany) equipped with a DiaTOME histo diamond knife (45degrees, 6 mm, MX559, Diatome AG, Nidau, Switzerland). Sections were stained with a solution containing 1% methylene blue (#881232022, Grüssing GmbH, Filsum, Germany), 1% Azure II (#861065-25G, Sigma-Aldrich, St. Louis, MO, USA), and 1% di-sodium tetraborate decahydrate (#CN07.1, Roth, Karlsruhe, Germany) in distilled water.

Images of the cervical spinal cord from both control and caffeine-treated wildtype and Wobbler mice were captured using a Keyence BZ-X800 (Keyence, Osaka, Japan). Overview images of the ventral horn were used to identify motor neurons based on specific morphological features, including size, presence of a distinct nucleus, and Nissl bodies. In accordance with established criteria from stereological and histological studies [57, 58] only large multipolar neurons located in lamina IX of the ventral horn with soma diameters of approximately 25–50 μm were included in the analysis, as these features are characteristic of alpha motor neurons and allow reliable distinction from interneurons or glial cells. High-magnification images were taken to analyze motor neuron morphometry using ImageJ 1.51s (National Institutes of Health, USA) software. The following parameters were evaluated: (i) number of motor neurons per ventral horn assessing neuronal density and (ii) area of the soma (µm^2^) reflecting body size. At least 10 ventral horns per group were analyzed for motor neuron quantification, and at least 100 motor neurons per group were measured for morphometric evaluation. The morphological analyses included 3 animals from each group.

### Statistical analysis

Statistical analyses were performed using GraphPad Prism 9.0 (GraphPad Software, San Diego, CA, USA). Data are presented as mean values ± standard error of the mean (SEM) and were derived from at least three independent experiments. Kolmogorov-Smirnov normality test was used to confirm normal distribution. A two-tailed Student’s t-test was used to evaluate differences between two groups. For comparisons involving multiple groups, a two-way analysis of variance (ANOVA) was performed, followed by Tukey’s post-hoc test for pairwise comparisons. A *p*-value < 0.05 wase considered statistically significant.

## Results

### Caffeine transiently preserves muscle strength in wobbler mice

**Fig. 2:**
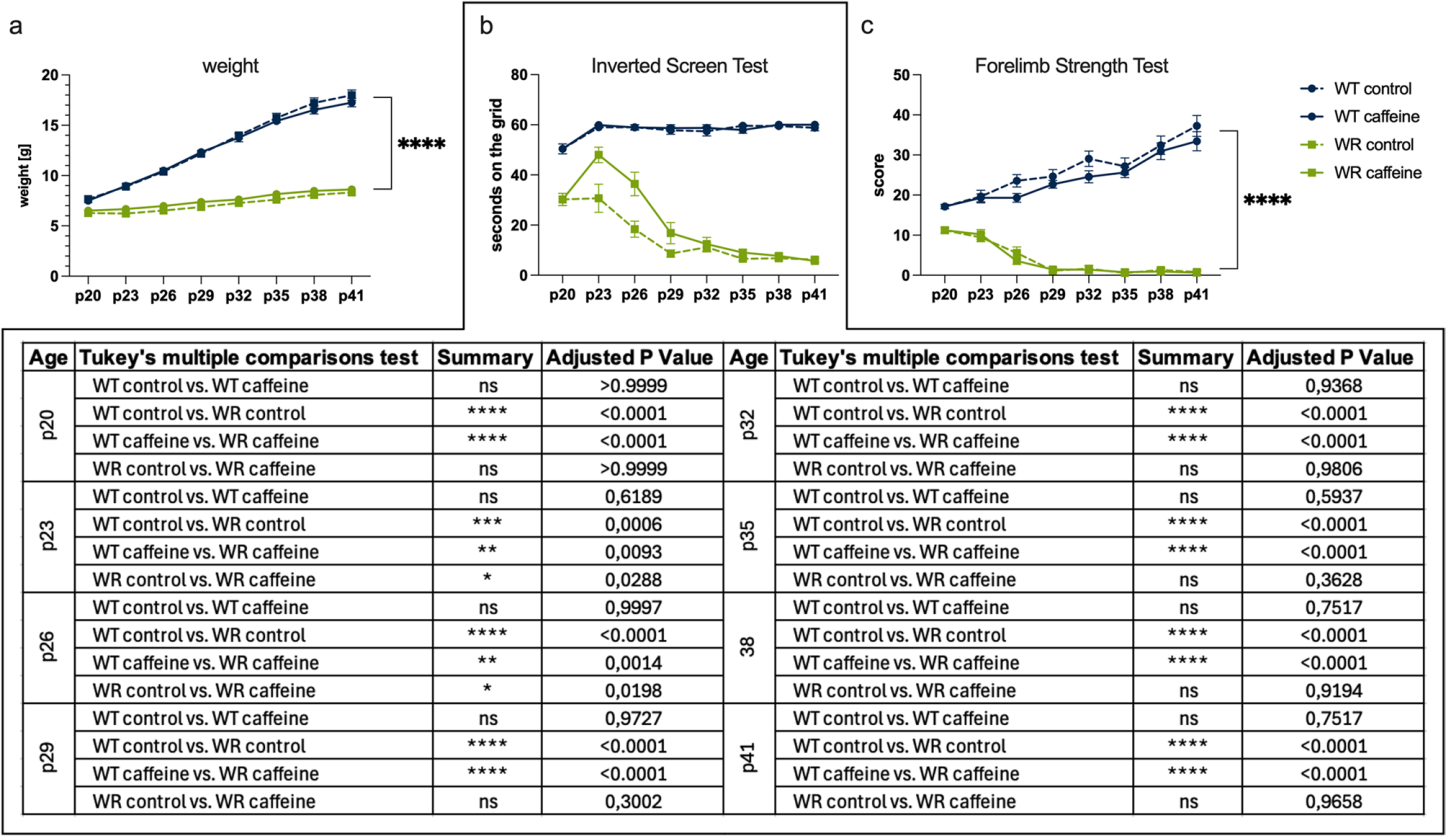
Impact of caffeine supplementation on body weight and muscle strength in wildtype (WT) and Wobbler (WR) mice. **a** Body weight of WT and WR mice over the course of the study (p20–p41). While WR mice exhibited significantly lower body weight compared to WT mice, caffeine supplementation had no effect on the weight of either genotype. For clarity of presentation, a single bracket summarizes the consistent and highly significant differences between WT and WR or WT (Caf) and WR (Caf) groups across all time points (****p < 0.0001). **b** Performance in the inverted screen test, assessing whole-body grip strength. WT and WT (Caf) mice maintained consistently high performance, holding onto the grid for 60 s from p23 onwards. WR mice showed a marked decline in grip strength between p20 and p29. Caffeine-treated WR mice (WR Caf) exhibited significantly improved performance compared to untreated WR mice at p23 and p26, but no differences were observed between these groups from p32 onwards. To maintain clarity, all relevant pairwise statistical comparisons, including differences between genotypes under identical treatment and treatment effects within the same genotype, are summarized in the table below the graph. **c** Forelimb strength test results. WT and WT (Caf) mice displayed a continuous increase in strength from p20 to p41, while WR and WR (Caf) mice showed a progressive decline, becoming unable to lift even the lightest weight from p29 onwards. No significant differences were observed between WR and WR (Caf) in this test. For clarity, a single summarizing bracket is shown on the right side of the graph to indicate that, at each analyzed time point, forelimb strength was significantly higher in WT compared to WR mice under the same treatment conditions (WT vs. WR and WT Caf vs. WR Caf; ****p < 0.0001). Data are expressed as mean ± SEM; *p < 0.05, **p < 0.01, ***p < 0.001, ****p < 0.0001 (2way ANOVA with Tukey’s multiple comparisons test). *n* = 16 per group

Body weight was monitored throughout the study period. As expected, WR mice exhibited significantly lower body weight compared to their WT littermates across all time points. However, caffeine supplementation had no discernible effect on body weight in either genotype (Fig. 2a). To assess the impact of caffeine on motor performance and muscle strength, two complementary tests were employed: the inverted screen test, which evaluates grip strength of all four limbs, and the forelimbs test, which measures forelimb-specific strength.

In the inverted screen test, WT and WT (Caf) mice showed almost identical performance. At p20, most WT mice held onto the grid for approximately 50 s, and from p23 to p41, they consistently maintained the full 60 s (Fig. 2b). In contrast, WR mice exhibited a marked decline in grip strength. Starting with a mean grip duration of around 30 s at p20, their performance decreased to approximately 20 s at p26 and dropped further to 10 s or less from p29 onwards (Fig. 2b). This rapid decline in motor performance between p20 and p29 was particularly notable. During this early symptomatic window, WR (Caf) mice demonstrated a transient, yet measurable, improvement: they held onto the grid for nearly 20 s longer at p23 and p26 compared to untreated WR mice, with a statistically significant difference. However, this benefit was not sustained beyond p26. from p32 onwards, WR (Caf) mice declined to levels comparable to untreated WR mice, with no discernible differences between the groups. These results suggest a short-term functional benefit of caffeine that transiently mitigates the early decline in muscle strength.

In the forelimbs test, WT and WT (Caf) mice showed a continuous improvement in strength over time (Fig. 2c). Their average score increased from around 18 at p20 to over 30 at p41, indicating steady muscle development. In contrast, neither WR nor WR (Caf) mice exhibited any strength gain (Fig. 2c). Both groups started with a mean score just above 10 at p20, which progressively declined to nearly zero by p29. From this point onwards, WR mice, regardless of treatment, were unable to lift even the lightest weight. Consequently, no significant differences were observed between WR and WR (Caf) mice in the forelimbs test.

### Caffeine improves NAD^+^ levels and ROS levels but does not affect NMNAT2 expression

To evaluate the effects of caffeine supplementation, three key parameters, (i) NMNAT2 expression, (ii) NAD levels, and (iii) ROS levels, were measured in the cervical spinal cord of the animals. Given the critical role of these factors in mitigating oxidative stress and supporting neuronal health, their analysis provides insights into the potential protective effects of caffeine in the ALS-like phenotype of Wobbler mice.

**Fig. 3:**
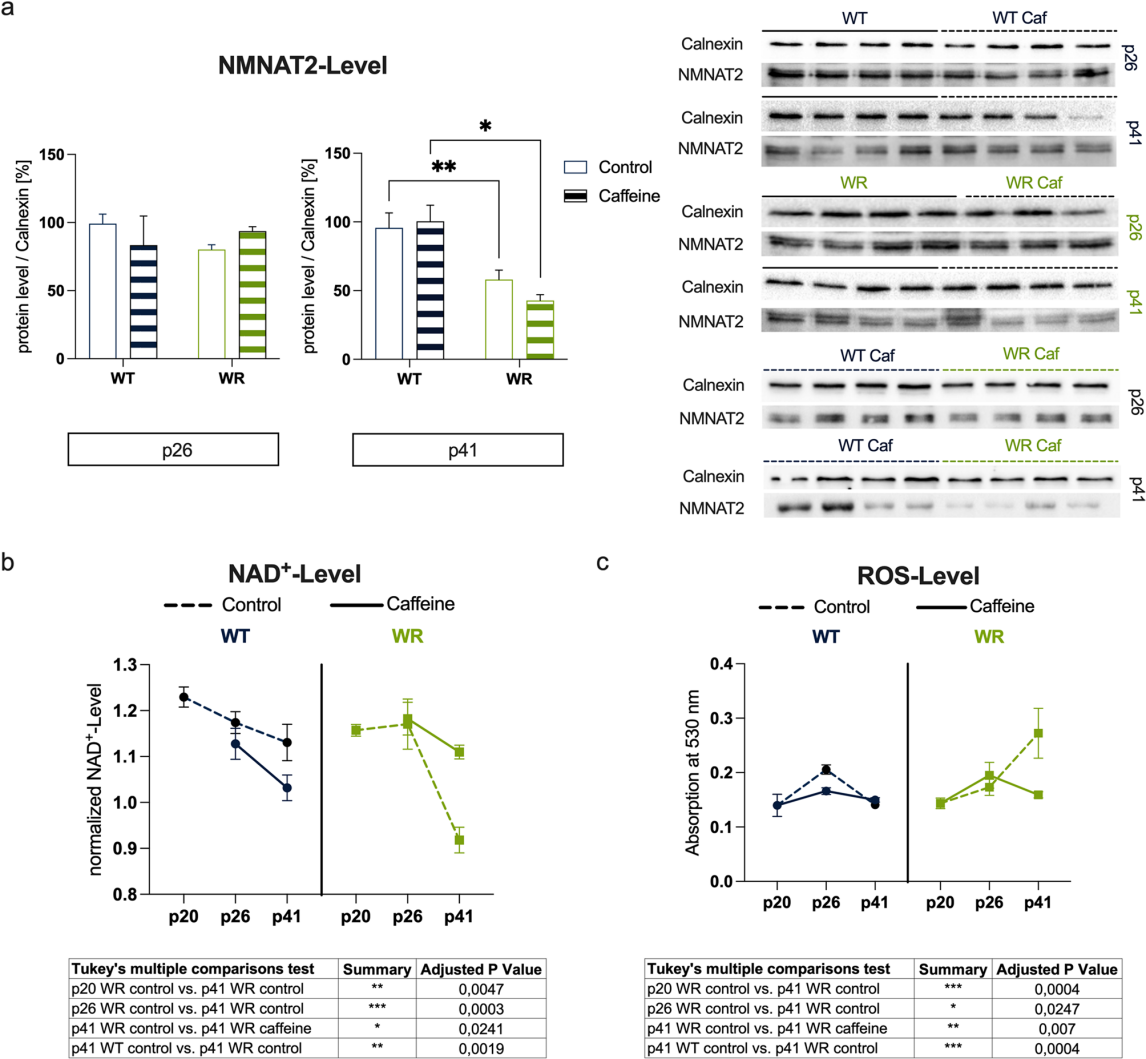
Effects of caffeine supplementation on NMNAT2 expression, NAD^+^ levels, and ROS levels in the cervical spinal cord of wildtype (WT) and Wobbler (WR) mice. **a** Semiquantitative analyses of NMNAT2 protein expression relative to calnexin at p26 and p41 as well as representative blots. No significant differences in NMNAT2 levels were observed across groups at p26. At p41, NMNAT2 expression was significantly reduced in WR compared to WT mice. Caffeine supplementation did not alter NMNAT2 levels in WR mice. **b** NAD⁺ levels at p20, p26, and p41. Both WT and WR mice showed a significant reduction in NAD⁺ levels from p20 to p41, with a markedly stronger decrease in WR mice. Caffeine supplementation effectively prevented NAD⁺ decline in WR mice, restoring levels at p41 to values comparable to WT. **c** ROS levels in the cervical spinal cord at p20, p26, and p41. At p20, ROS levels were comparable across all groups. By p41, untreated WR mice exhibited a significant increase in ROS levels compared to p20 values, while WR (Caf) mice showed significantly lower ROS levels, indicating an antioxidative effect of caffeine. To improve clarity, all relevant statistically significant pairwise comparisons and adjusted *p*-values are summarized in the tables below panels (**b**) and (**c**). Data are expressed as mean ± SEM; *p < 0.05, **p < 0.01, ***p < 0.001 (2way ANOVA with Tukey’s multiple comparisons test). *n* = 4 per group

NMNAT2 protein expression, previously shown to be influenced by caffeine at the mRNA level in motor neurons ex vivo [46], was analyzed in vivo using calnexin as a loading control. At p26, no significant differences in NMNAT2 protein levels were observed across groups, with both WT and WR animals displaying comparable expression regardless of treatment (Fig. 3a). However, by p41, a significant reduction in NMNAT2 expression was observed in WR compared to age-matched WT mice. Importantly, caffeine supplementation did not affect NMNAT2 expression in the spinal cord of the animals, as no significant differences were detected between WR and WR (Caf) groups (Fig. 3a).

Given the functional interplay between NMNAT2 and NAD^+^ metabolism, NAD^+^ levels were assessed to determine whether caffeine could modulate this critical metabolite. At p20 and p26, no significant differences in NAD^+^ levels were observed between WT and WR mice, indicating that NAD^+^ levels remain stable during these early stages of disease development, regardless of genotype (Fig. 3b). Furthermore, caffeine supplementation did not alter NAD^+^ levels in either genotype at these time points. By p41, significant differences in NAD^+^ levels emerged between untreated WT and WR mice. WR animals exhibited a substantial reduction in NAD^+^ levels in the cervical spinal cord compared to WT controls. However, caffeine supplementation prevented this decline, as NAD^+^ levels in WR (Caf) mice were restored to levels comparable to WT animals, effectively eliminating the genotype-dependent difference observed in untreated groups (Fig. 3b).

As NAD^+^ plays a central role in mitigating ROS formation, the influence of caffeine supplementation on ROS levels was also investigated. ROS levels showed characteristic differences across groups and age stages (Fig. 3c). In WT animals, ROS levels remained statistically unchanged across p20, p26, and p41, regardless of caffeine treatment. In WR mice, ROS levels at p26 were still comparable to those at p20, indicating no significant increase during this early phase. However, by p41, untreated WR mice exhibited a significant elevation in ROS levels compared to both p20 and p26. Notably, caffeine supplementation mitigated this effect, as ROS levels in WR (Caf) mice at p41 were significantly lower than in untreated WR animals, indicating a robust antioxidative effect of caffeine.

### Caffeine reduces motor neuron loss at p41 but does not influence morphology

**Fig. 4:**
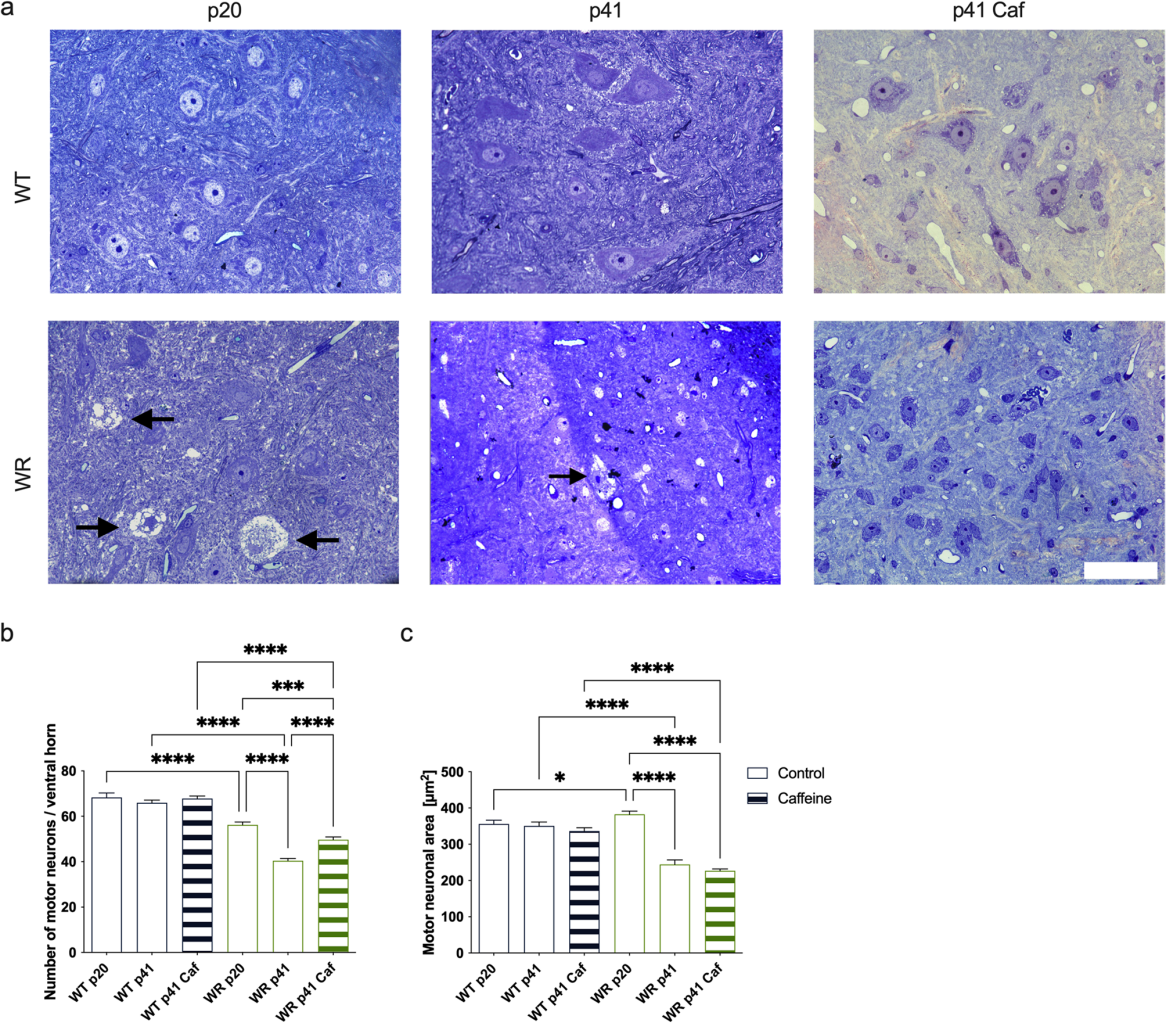
Morphological analysis of motor neurons in the cervical spinal cord of WT and WR mice. **a** Representative semi-thin sections of the ventral horn in the cervical spinal cord at p20 and p41, showing differences in motor neuron morphology across groups. In WR mice at p20, arrows indicate degenerating motor neurons. Scale bar: 50 μm (applies to all images). **b** Quantification of motor neuron counts per ventral horn. WR mice exhibited significantly fewer motor neurons compared to WT mice at both p20 and p41. Between p20 and p41, untreated WR mice showed a marked decline in motor neuron numbers, while WR (Caf) mice displayed a highly significant increase in motor neuron counts at p41 compared to untreated WR mice, suggesting a protective effect of caffeine. **c** Analysis of soma area. WT mice maintained consistent soma areas across all time points, whereas WR mice displayed significantly enlarged soma at p20, indicative of pathological swelling. By p41, soma areas in WR mice had significantly decreased compared to WT. Caffeine supplementation did not alter soma area in WR mice. Data represent mean values calculated per animal from > 100 motor neurons per group (*n* = 3 animals per group). Statistical comparisons were performed using these biological replicates; *p < 0.05, ***p < 0.001, ****p < 0.0001 (2way ANOVA with Tukey’s multiple comparisons test). *N* = 3 per genotype, n = > 100 motor neurons per group

To complement the phenotypic and biochemical analyses, the morphology of motor neurons in the cervical spinal cord was evaluated to provide histological insights into the effects of caffeine supplementation. Semi-thin sections were analyzed to determine motor neuron counts per ventral horn, as well as soma area, to assess pathological changes at the cellular level and potential parallels with caffeine treatment (Fig. 4). The number of motor neurons per ventral horn was significantly reduced in WR mice compared to WT mice at both p20 and p41 (Fig. 4b). Between p20 and p41, WR mice exhibited a marked loss of motor neurons, with approximately 20 fewer neurons per ventral horn, while WT mice maintained stable neuron counts over the same period. Importantly, caffeine supplementation resulted in a highly significant increase in the number of motor neurons in WR (Caf) mice at p41 compared to untreated WR animals, suggesting a protective effect of caffeine against motor neuron loss (Fig. 4b). The analysis of soma area revealed distinct differences between WT and WR groups. WT mice showed consistent soma areas across all time points and treatments (Fig. 4c). In contrast, WR mice displayed significantly enlarged motor neuronal soma at p20, indicative of pathological swelling (Fig. 4a, c). By p41, the soma area in WR mice had decreased significantly, resulting in smaller soma sizes compared to WT mice. Notably, caffeine supplementation did not influence the soma area in WR mice, as no significant differences were observed between WR and WR (Caf) groups (Fig. 4c). Overall, caffeine supplementation demonstrated a protective effect by preserving higher motor neuron counts in WR mice at p41. However, since the analysis was limited to this time point, we cannot exclude the possibility that motor neuron loss may have been merely delayed rather than prevented. Therefore, our findings should be interpreted as a temporal attenuation of motor neuron degeneration. Notably, caffeine did not influence changes in soma area, which reflect pathological processes associated with the progression of the ALS-like phenotype in Wobbler mice. These findings highlight the potential of caffeine to reduce motor neuron loss without altering soma morphology in the considered period up to p41.

## Discussion

Oxidative stress is a hallmark of ALS pathogenesis, characterized by elevated ROS levels and damage to cellular components such as lipids, proteins, and DNA [9–12]. These pathological features are consistently observed in both ALS patients and preclinical models, including the Wobbler mouse, which serves as a valuable tool for investigating potential therapeutic interventions targeting oxidative stress [13–18, 27, 28]. This study evaluated the efficacy of caffeine, a compound with well-documented antioxidant properties [34, 35], in mitigating motor neuron degeneration and its associated biochemical and morphological changes in Wobbler mice. Although previous studies suggested beneficial roles of caffeine in neurodegeneration, including Parkinson’s and ALS-like models, these primarily focused on in vitro data or endpoint analyses [46, 59, 60]. In contrast, our study provides an age-specific in vivo assessment in the Wobbler model, enabling a temporal perspective on caffeine’s effects during disease progression. It was specifically designed to evaluate whether caffeine’s previously reported ability to upregulate Nmnat2 in vitro [45, 46, 61] would translate into a measurable in vivo effect. By linking functional outcomes with molecular readouts such as ROS and NAD⁺ levels, we offer new evidence supporting the potential of caffeine as a modulator of early disease processes, even though the precise underlying mechanisms remain to be elucidated.

The motor tests using the inverted screen and forelimb strength tests demonstrated that caffeine supplementation transiently improved motor performance during the early stages of disease progression. WR (Caf) mice showed significant improvements in grip strength of all four limbs at p23 and p26 compared to untreated WR mice, indicating a short-term functional benefit. However, this effect diminished starting from p32, consistent with the observation that cervical motor neurons, likely responsible for forelimb strength, were already severely compromised and could not be rescued by caffeine. This discrepancy may reflect differences in the use of body regions not specifically assessed in the forelimb test. In video recordings, WR (Caf) mice frequently adopted a curled body posture while gripping the grid, suggesting a possible compensatory stabilization through trunk musculature. In contrast, untreated WR mice tended to stretch out and lost grip more rapidly. Although these observations were not quantitatively analyzed, they may point to subtle motor compensation strategies that warrant further investigation.

A limitation of our study is the absence of a direct mechanistic link between caffeine and improved muscle strength. The functional benefits observed at p23 and p26 may reflect early metabolic support or non-neuronal compensatory mechanisms, rather than direct protection of vulnerable motor neurons. Since NMNAT2 expression was unchanged and no morphological differences in soma were observed, future studies should include analyses of neuromuscular junction integrity and muscle tissue to establish a causal relationship between biochemical improvements and motor function.

The discrepancy between early functional improvements and the delayed biochemical effects observed at p41 likely reflects the multiphasic and region-specific nature of ALS pathology. While motor performance benefits were not sustained beyond p26, the preserved NAD⁺ levels and reduced ROS at p41 indicate ongoing molecular protection, which may contribute to the increased survival of motor neurons observed histologically. This highlights the complexity of therapeutic timing and suggests that caffeine acts through temporally distinct but complementary pathways. The preservation of motor neuron numbers at p41 likely reflects earlier protective influences that coincided with the transient functional improvements observed at p23–p26.

Histological analyses further supported these findings, revealing a higher number of surviving motor neurons in the cervical spinal cord of WR (Caf) mice at p41 compared to untreated WR mice. These results suggest that caffeine treatment mitigates motor neuron loss during early symptomatic stages. However, due to the lack of data beyond p41, it remains unclear whether this effect reflects a sustained influence on disease progression or merely a transient delay in degeneration. Longitudinal studies including later disease stages are necessary to clarify the durability of this neuroprotective effect.

Importantly, caffeine treatment in this study was deliberately initiated at postnatal day 20 (p20), a time point when cervical motor neuron degeneration is already ongoing in the Wobbler mouse. This approach was chosen to reflect a clinically relevant situation in which therapeutic interventions typically begin only after symptom onset and diagnosis in ALS patients. Our aim was therefore not to assess preventive effects, but to determine whether caffeine can modulate disease progression once neurodegeneration has commenced. Accordingly, our results do not allow conclusions about a delay in disease onset. Nevertheless, the observed histological and biochemical findings suggest that caffeine may exert supportive effects even when administered after the onset of neurodegeneration.

While most previous studies suggest beneficial effects of caffeine in models of neurodegeneration, its role in ALS remains controversial [62]. Notably, a study by Potenza et al. (2013) reported that chronic caffeine intake significantly shortened survival and accelerated disease progression in SOD1-G93A mice, a widely used transgenic model of ALS [52]. This discrepancy may reflect fundamental differences between ALS models: SOD1-G93A mice exhibit a transgene-driven disease course with later symptom onset (typically around postnatal day 90) and progressive neurodegeneration [63], along with a marked downregulation of adenosine A2A-receptors in the spinal cord [52]. In contrast, Wobbler mice develop ALS-like symptoms already around postnatal day 20 and represent a non-transgenic, developmentally earlier-onset model [26]. Under these differing conditions, additional receptor antagonism by caffeine may exacerbate disease in SOD1-G93A mice, while in Wobbler mice it may interact more favorably with early pathophysiological processes. While direct data on adenosine signaling in Wobbler mice are currently lacking, it is conceivable that differences in receptor expression or downstream pathways may contribute to the divergent effects of caffeine. Moreover, the timing of caffeine administration may also play a role. In our study, treatment was initiated at symptom onset (p20), whereas Potenza et al. began caffeine exposure pre-symptomatically, which may have interacted differently with stress and repair pathways. Finally, a recent expert review concluded that the current clinical and preclinical data on caffeine in ALS are inconclusive and insufficient to support its therapeutic use in patients [64]. These observations underline the importance of model-specific interpretation and the need for further comparative studies to determine under which conditions caffeine may be protective or detrimental in ALS.

In addition to its antioxidant properties, caffeine may exert neuroprotective effects through modulation of neuroinflammation. As a non-selective antagonist of adenosine receptors, caffeine particularly targets A2A-receptors, which are abundantly expressed in microglia and astrocytes and are implicated in the regulation of inflammatory responses in the CNS [65]. Antagonism of A2A-receptors has been shown to reduce microglial activation, inhibit pro-inflammatory cytokine release, and mitigate excitotoxicity in various neurodegenerative disease models, including Alzheimer’s and Parkinson’s disease [65, 66]. While direct evidence for these mechanisms in ALS and specifically in the Wobbler mouse is lacking, it is conceivable that caffeine’s interaction with adenosine signaling may attenuate neuroinflammatory processes also in this context. Thus, the observed protective effects of caffeine on motor neurons may reflect a multifactorial mechanism involving both redox balance and inflammation control. Supporting this hypothesis, additional Western blot analyses of spinal cord lysates revealed that pro-Caspase-1 remained elevated in WR (Caf) mice, while the previously observed increases in mature IL-1β and IL-18 [67] were no longer detectable following caffeine supplementation at p41. This pattern may indicate that inflammasome activation was attenuated, potentially contributing to reduced neuroinflammation. The corresponding data are provided in Supplementary Figure S2. Future studies should explore this possibility by assessing microglial and astroglial activation in the Wobbler spinal cord and their modulation by caffeine.

Although caffeine did not influence the characteristic hypertrophy at p20 or subsequent somatic shrinkage at p41, its neuroprotective effect was reflected in the preservation of motor neuron counts at p41. These findings raise the question of whether soma size is a reliable marker of vulnerability in ALS. In fact, several studies suggest that soma size plasticity, encompassing both hypertrophy and shrinkage, may reflect dynamic stress responses rather than predict neuronal survival. For example, Fayad et al. (2013) demonstrated in the SOD1-G93A mouse model that motoneurons of varying sizes can undergo degeneration [68]. Similar findings have been made in other ALS models and in human patients, where somatic shrinkage is a hallmark of advanced disease stages [68, 69]. This differential effect mirrors the clinical progression of ALS in humans, where the disease often starts in one region and spreads to adjacent areas over time. Notably, 36–67% of ALS cases begin with upper limb involvement [70, 71], typically progressing to contiguous body regions rather than the bulbar region [72, 73]. The protective effects of caffeine on probably unaffected motor neurons, i.e. neuronal populations outside the already affected cervical region, could suggest its potential as an adjunct therapy in the early stages of ALS, particularly for slowing the spread of neurodegeneration.

The well-documented ability of caffeine to inhibit lipid peroxidation and reduce ROS production [44, 74] provided the rational for its use in this study. Regular caffeine consumption has been shown to alleviate oxidative stress and enhance mitochondrial function in various neurotoxic conditions [75]. Consistent with prior findings, our results demonstrated that caffeine supplementation significantly reduced superoxide anion levels in the cervical spinal cord of WR mice compared to untreated controls. The reduction of ROS levels is hypothesized to contribute to downstream protective effects by preserving NAD^+^ levels [45] and mitigating neuronal damage.

WR (Caf) mice at p41 exhibited significantly restored NAD^+^ levels in the cervical spinal cord compared to untreated WR mice, reaching values comparable to WT controls. These findings align with previous studies demonstrating reduced NAD^+^ levels in WR mice during advanced disease stages [27]. Notably, this effect occurred independently of NMNAT2 expression, as caffeine supplementation did not increase NMNAT2 protein levels in vivo. While NMNAT2 is a critical enzyme for NAD^+^ synthesis, its expression remained significantly reduced in WR mice at p41, regardless of caffeine treatment. In contrast, in vitro studies have reported an upregulation of NMNAT2 mRNA following caffeine treatment [46], an effect that was also observed in vivo in a different neurodegenerative model, where caffeine restored NMNAT2 levels in rTg4510 tauopathy mice [45]. The lack of NMNAT2 induction in Wobbler mice suggests that tissue-specific regulatory mechanisms or disease-specific factors might influence the response to caffeine. Additionally, although prior research suggests minimal differences in oral versus intraperitoneal caffeine administration in mice [76], variations in pharmacokinetics or bioavailability could still play a role. Although our study did not include measurements of classical antioxidant enzymes such as SOD or CAT, future investigations should consider their involvement, particularly in light of established links between caffeine, NRF2 activation, and endogenous antioxidant defense systems such as the glutathione system [28, 77–81]. It remains possible that caffeine’s protective effects result from the convergence of multiple pathways, including redox modulation, inflammation control, and preservation of NAD⁺ levels, rather than a single mechanism. As such, our findings contribute to refining the understanding of caffeine’s multifaceted action profile in ALS.

**Fig. 5:**
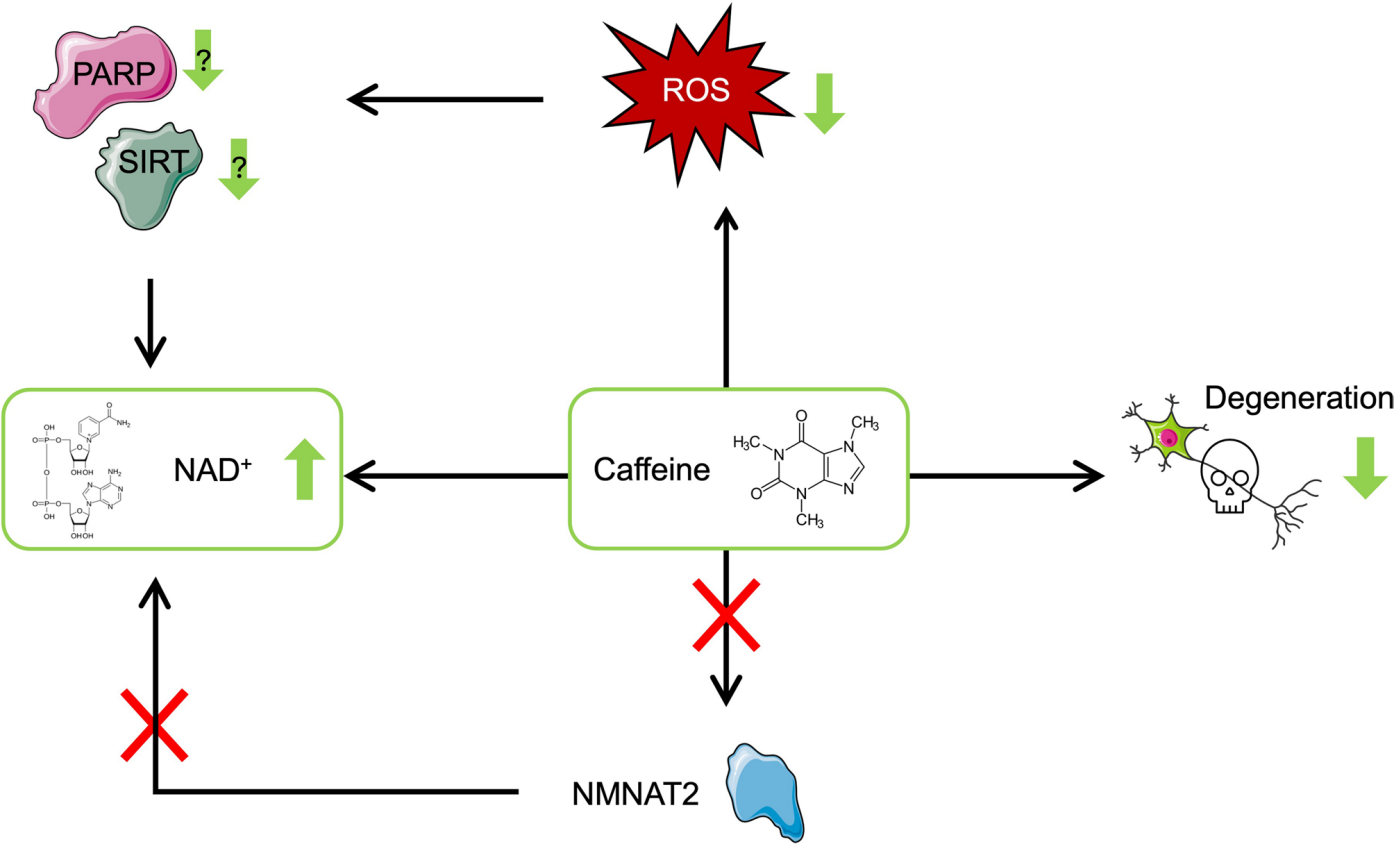
Hypothetical model of caffeine’s neuroprotective mechanism in Wobbler mice. Caffeine supplementation reduces reactive oxygen species (ROS) levels, potentially leading to decreased activation of NAD^+^-consuming enzymes, such as Poly-ADP-Ribose Polymerases (PARPs) and Sirtuins (SIRTs). This reduction may contribute to the observed preservation of NAD^+^ levels. The increase in NAD^+^ occurs independently of NMNAT2 expression, as caffeine did not enhance NMNAT2 levels in this study. The antioxidant effects of caffeine, together with its impact on NAD metabolism, might mitigate neuronal degeneration, delaying disease progression. Green arrows indicate presumed beneficial effects (reduction of ROS and neuronal degeneration, increase in NAD^+^), while red X-marks highlight the lack of direct NMNAT2 involvement. This figure represents a working model based on observed NAD^+^ preservation and known literature. The actual involvement of PARPs and SIRTs remains to be experimentally confirmed

Since NMNAT2 expression was not altered by caffeine supplementation, the observed increase in NAD^+^ levels likely results from alternative regulatory mechanisms. Given the well-established role of NAD-consuming enzymes in neurodegeneration, a plausible hypothesis is that caffeine influences NAD^+^ availability by modulating these pathways. Specifically, caffeine may reduce the activation of NAD-consuming enzymes, such as Poly-ADP-Ribose Polymerases (PARPs) and Sirtuins (SIRTs), due to its antioxidant properties. By lowering ROS levels, the demand for DNA repair and other NAD-consuming processes might decrease, preserving NAD^+^ levels. Figure 5 illustrates this hypothesized mechanism, where caffeine supplementation reduces ROS, consequently lowering the activity of NAD-consuming enzymes and preserving NAD^+^ levels. This mechanism appears to occur independently of NMNAT2 expression, as shown in this study. We emphasize that this interpretation remains speculative. Although consistent with existing literature on the roles of PARPs and SIRTs in oxidative stress responses, our data do not directly validate this pathway. Future mechanistic studies involving activity assays or transcript/protein level assessments of NAD-consuming enzymes will be necessary to confirm this proposed mechanism.

This hypothesis is further supported by previous evidence. As the disease progresses, NAD-consuming processes, such as those mediated by Poly-ADP-Ribose Polymerase 1 (PARP1), likely contribute to the significant decline in NAD levels observed at p41. PARP1, activated by oxidative stress-induced DNA damage, consumes NAD to facilitate DNA repair and contributes to neuronal death through PAR-dependent mechanisms, including parthanatosis [82, 83]. This is supported by the observation of significantly elevated γ-H2AX foci, indicative of increased DNA double-strand breaks (DSBs), in WR mice at p40 [28]. Notably, these DNA-DSBs could be mitigated by the administration of glutathione precursors or MitoTEMPO, a mitochondrial-targeted antioxidant, strongly suggesting oxidative stress as a key driver of DNA damage in this model.

Moreover, PARP-1 also promotes the formation of TDP-43 and FUS-containing granules in the cytoplasm, two RNA-binding proteins that appear as neuronal cytoplasmic inclusions in neurodegenerative diseases such as ALS [84]. Additionally, the interplay between NAD metabolism and sirtuins, NAD-dependent deacetylases involved in DNA repair and neuroprotection, represents another potential avenue for therapeutic intervention in ALS [85]. Sirtuins have been considered as disease-modifying factors in ALS for many years. In recent studies, positive effects on neuroprotection and survival have been investigated by manipulating sirtuin activities and concentrations in ALS models, with promising results especially for SOD1 mutations [86]. No studies on ALS based on sirtuin or sirtuin activators have yet been reported.

Epidemiological studies linking increased caffeine consumption to a reduced risk of neurodegenerative diseases, such as Parkinson’s disease [87], further support its potential as a therapeutic agent. There is evidence that both motor and cognitive symptoms can be alleviated. Caffeine modulates the adenosine A2A receptor, along with its effects on neuroinflammation, autophagy, and mitochondrial function, underpinning its neuroprotective properties [87]. Istradefylline is a caffeine analog that acts as an antagonist of the A2A adenosine receptor. It is used in the USA as an add-on therapy to levodopa/carbidopa in patients with Parkinson’s syndrome [87].

Clinical trials have explored the potential therapeutic effects of caffeine in neurodegenerative diseases. For instance, preliminary results from a clinical trial involving multiple sclerosis patients demonstrated that a daily dose of 200 mg of caffeine improved balance, functional mobility and quality of life [88]. This simple and cost-effective intervention showed the best results in patients with a moderate level of disability on the Expanded Disability Status Scale (EDSS) [89]. Despite these promising findings, the current evidence on caffeine’s efficacy in multiple sclerosis remains limited and heterogeneous, precluding meaningful meta-analysis [90].

Regarding safety, a review concluded that a daily caffeine intake of ≤ 400 mg (approximately 6.5 mg/kg body weight for a 70 kg person) is generally considered safe, with no significant adverse effects reported in healthy adults [91]. However, individual thresholds for caffeine tolerance vary based on weight and sensitivity, with symptoms of overdose including palpitations, anxiety, restlessness and tremor [92, 93]. While the acute toxic threshold for caffein is challenging to define, it is estimated to be around 10 g per day, equivalent to the caffeine contained in 100 cups of coffee [91]. These findings underscore the need for further research to establish the optimal dosing of caffeine in neurodegenerative diseases such as ALS, weighing its potential therapeutic benefits against the risks of adverse effects.

Although caffeine treatment resulted in transient improvements in grip strength, the precise mechanisms underlying this effect remain unresolved. These functional benefits may reflect early metabolic support or subtle compensatory strategies, as suggested by video observations of altered posture and increased trunk stabilization in WR (Caf) mice. Given that NMNAT2 expression and soma morphology were unaffected, it is unlikely that these factors account for the observed improvement. Interestingly, supplementary immunoblot analyses suggest that caffeine may also exert anti-inflammatory effects, as levels of mature IL-1β and IL-18 were not significantly elevated in WR (Caf) mice, despite continued presence of pro-Caspase-1. This may indicate attenuated inflammasome activation and reduced neuroinflammatory signaling. Future investigations should therefore not only examine neuromuscular junction integrity and muscle tissue, but also further explore the potential interplay between caffeine’s peripheral and central effects on inflammation and neuronal function.

### Study limitations and future directions

While our study provides important insights into the antioxidant and neuroprotective effects of caffeine in the Wobbler mouse, several limitations should be acknowledged. We deliberately focused on NAD⁺ homeostasis via NMNAT2, and although caffeine restored NAD⁺ levels, the precise molecular pathway remains unresolved. Functional benefits were observed transiently, and we cannot exclude contributions from neuromuscular junction integrity or compensatory motor strategies, which were not assessed. Moreover, our analyses were restricted to early disease stages (p20–p41), preventing conclusions about long-term effects or sustained neuroprotection. Finally, while we provide first evidence for attenuated inflammasome activation (reduced IL-1β and IL-18 processing), broader analyses of glial responses and classical antioxidant pathways are required to clarify the mechanisms through which caffeine exerts its effects. These limitations highlight the complexity of ALS pathophysiology and underscore the need for future mechanistic studies across different disease stages and models.

## Conclusion

This study demonstrates that caffeine supplementation exerts stage-specific neuroprotective effects in the Wobbler mouse model of ALS. While our initial focus was on NMNAT2 as a candidate mediator of caffeine’s effects, our findings show that neuroprotection occurred independently of NMNAT2 modulation. This suggests that other pathways, such as the regulation of ROS levels, NAD⁺ preservation, and attenuation of inflammatory signaling, may play a more central role. When administered from early symptomatic onset, caffeine transiently improved motor performance and mitigated motor neuron loss in the cervical spinal cord. Although the functional benefits diminished at later time points, biochemical analyses revealed sustained antioxidant effects, including reduced ROS levels and restored NAD⁺ availability, independent of NMNAT2 expression. Furthermore, caffeine treatment was associated with a lack of significant elevation in mature IL-1β and IL-18, suggesting a potential attenuation of neuroinflammatory signaling. While the precise molecular pathways remain to be elucidated, we propose that caffeine may act via multiple converging mechanisms, reducing oxidative stress, modulating NAD⁺ metabolism, and limiting inflammation, to delay neurodegeneration. These findings position caffeine as a promising, low-cost candidate for adjunctive therapy in ALS, although confirmation in extended longitudinal studies and mechanistic analyses will be necessary before translation into clinical application.

## Supplementary Information

Supplementary Material 1.

## Data Availability

All data supporting the findings of this study are included within the manuscript or supplementary information files.

## Abbreviations

ALS: Amyotrophic lateral sclerosis
Caf: Caffeine
DNA: Deoxyribonucleic acid
DSBs: Double–strand breaks
EDSS: Expanded disability status scale
EU: European union
FUS: Fused in sarcoma
GARP: Golgi–associated retrograde protein
IP: Intraperitoneal
MIC: Medical imaging center
mRNA: Messenger ribonucleic acid
NAD: Nicotinamide adenine dinucleotide
NADH: Nicotinamide adenine dinucleotide (Reduced Form)
NMNAT: Nicotinamide mononucleotide adenylyltransferase
PARP: Poly–ADP–ribose polymerase
PBS: Phosphate–buffered saline
PO: Per Os (Oral Administration)
ROS: Reactive oxygen species
SEM: Standard error of the mean
SIRTs: Sirtuins
SOD1: Superoxide dismutase 1
TDP: 43–Transactive response DNA binding protein 43 kDa
Vps54: Vacuolar protein sorting–associated protein 54
WT: Wildtype
WR: Wobbler
