# Supplementary material for: Caffeine mitigates ROS accumulation and attenuates motor neuron degeneration in the wobbler mouse model of amyotrophic lateral sclerosis
Source: Cell Commun Signal. 2025 Sep 10;23:394. doi: 10.1186/s12964-025-02415-5 (PMC12421768; doi:10.1186/s12964-025-02415-5)
Supplement: Supplementary file 1 — Supplementary Material 1. [file 12964_2025_2415_MOESM1_ESM.pdf]

Supplementary File:

Supplementary Table S1: Estimated body weight, water intake, and resulting caffeine dose range in wild-type (WT) and Wobbler (WR) mice during the treatment period (p20–p40). Body weights and water intake estimates were derived from prior in-house measurements and published averages for mice of comparable strains. Drinking volumes were assumed to range between 5–7 ml per 30 g body weight per day. Caffeine was administered via drinking water at a concentration of 0.36 mg/ml. The table shows the corresponding range of estimated caffeine doses (in mg/kg/day), based on minimum and maximum water intake at each time point. These estimates demonstrate that the intended dose of 60 mg/kg/day was consistently achieved or exceeded across all ages and genotypes.

**Postnatal Day**	**WT Body Weight (g)**	**WT Water Intake (ml/day)**	**WT estimated Caffeine Dose Range (mg/kg/day)**	**WR Body Weight (g)**	**WR Water Intake (ml/day)**	**WR estimated Caffeine Dose Range (mg/kg/day)**
p20	6	1.0-1.4	60-84	6	1.0-1.4	60-84
p25	10	1.7-2.3	61.2-82.8	7,5	1.3-1.6	62.4-76.8
p30	11	1.8-2.6	58.9-85.1	9	1.5-2.1	60-84
p35	14	2.3-3.3	59.1-84.9	10	1.7-2.3	61.2-82.8
p40	16	1.8-2.5	60.8-83.2	10,5	1.8-2.5	61.7-85.7

Full uncropped Blots for Figure 3a

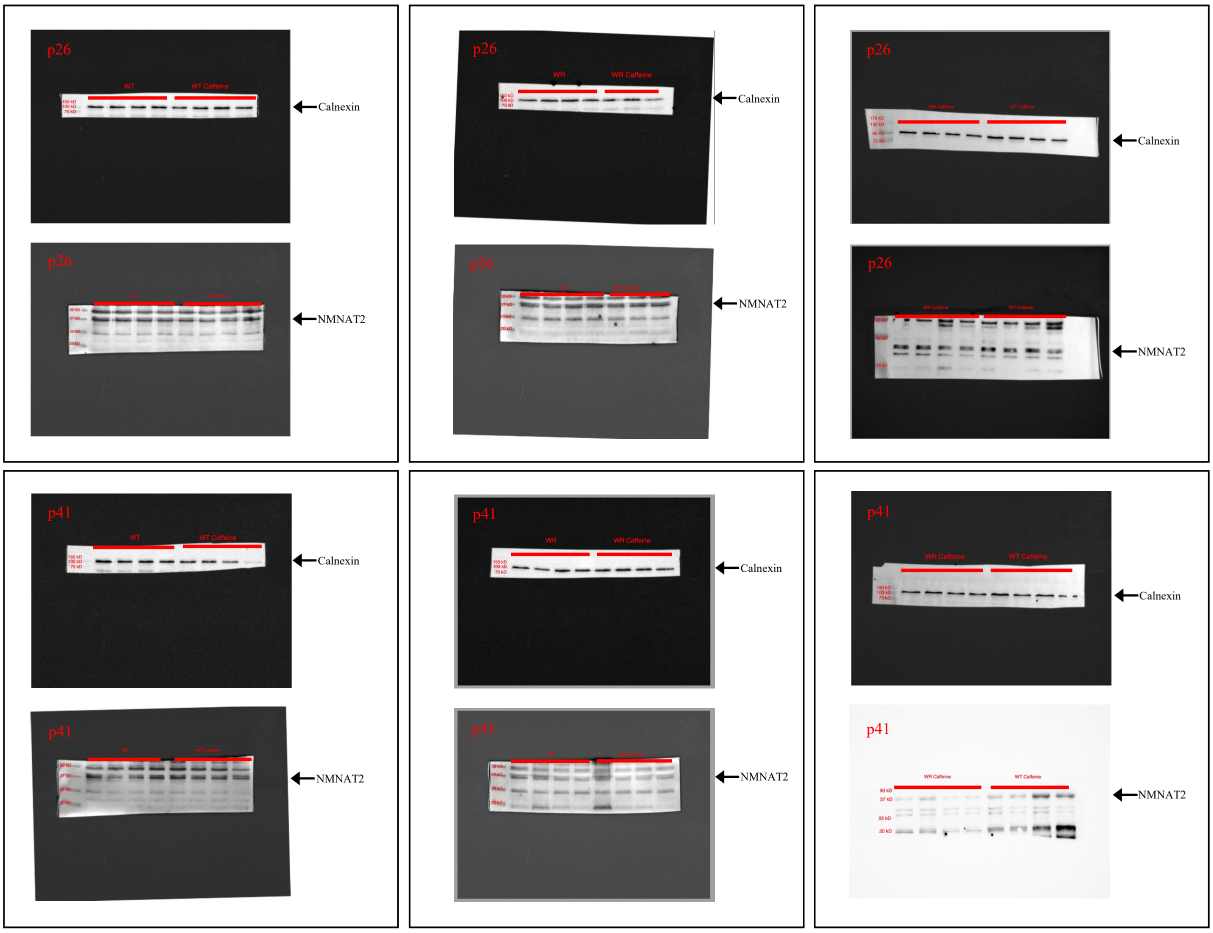

Supplementary Figure 1: **Full-length, uncropped Western Blots corresponding to Figure 3a.** Full Western Blot images for NMNAT2 and Calnexin protein detection in cervical spinal cord samples of wildtype (WT) and Wobbler (WR) mice at p26 and p41. Calnexin was used as a loading control. The blots correspond to the quantifications shown in Figure 3a.

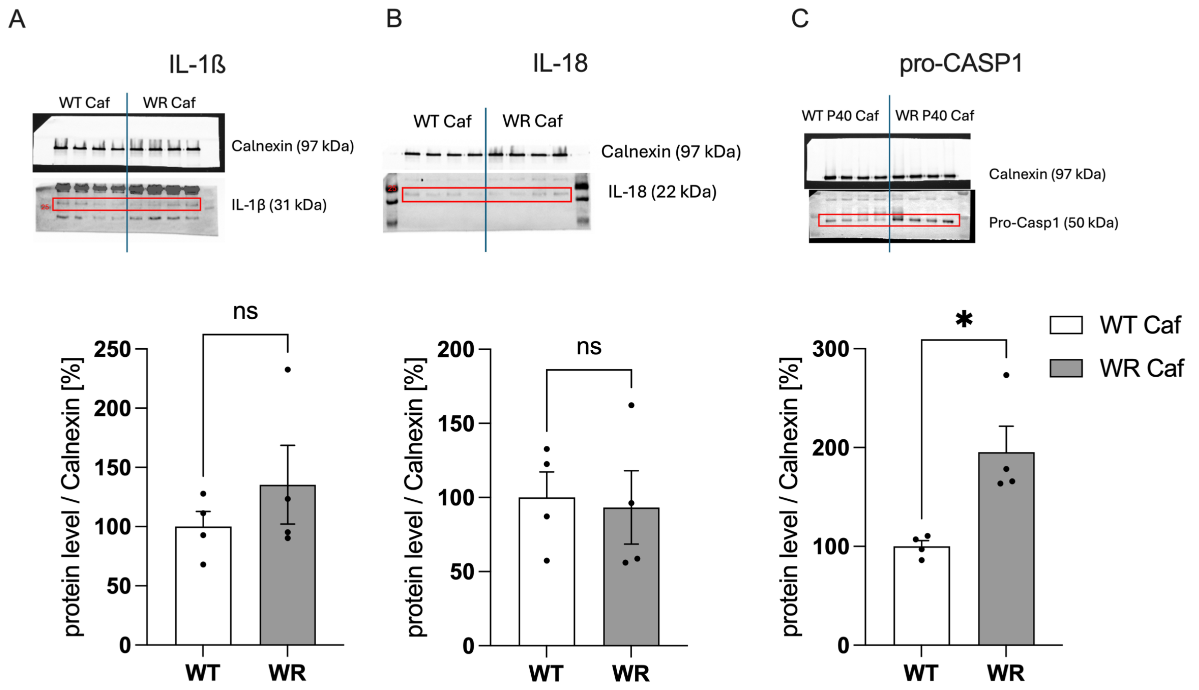

Supplementary Figure 2. Expression of inflammasome-related proteins in the spinal cord of caffeine-treated wild-type and Wobbler mice. Western blot analysis and densitometric quantification of (A) IL-1β, (B) IL-18, and (C) pro-Caspase-1 in cervical spinal cord lysates from caffeine-treated wild-type (WT) and Wobbler (WR) mice at postnatal day 41 (p41). Calnexin was used as loading control. No significant differences were observed in IL-1β and IL-18 levels between WT Caf and WR Caf animals. In contrast, pro-Caspase-1 expression remained significantly elevated in WR Caf mice. Untreated samples have been reported previously (Cihankaya et al., 2024). Data are presented as mean ± SEM. *$p < 0.05$; ns: not significant. N=4.
